# Levels of exhaled carbon monoxide measured during an intervention program predict 1-year smoking cessation: a retrospective observational cohort study

**DOI:** 10.1038/s41533-017-0060-8

**Published:** 2017-10-16

**Authors:** Huei-Guan Shie, Sheng-Wei Pan, Wen-Kuang Yu, Wei-Chih Chen, Li-Ing Ho, Hsin-Kuo Ko

**Affiliations:** 1 Division of Respiratory therapy, Department of Chest Medicine, Taipei Veterans General Hospital, No. 201, Sec. 2, Shih-Pai Road, Taipei 112, Taiwan Republic of China; 2 School of Respiratory Therapy, Taipei Medical University, Taipei, Taiwan Republic of China; 3 Division of Pulmonary Medicine, Department of Internal Medicine, Wan Fang Hospital, Taipei Medical University, Taipei, Taiwan Republic of China; 4 School of Medicine, National Yang-Ming University, Taipei, Taiwan Republic of China

## Abstract

Life-long smoking cessation is a critical public health objective, but it is difficult for numerous people. This study aimed to identify the independent predictors of 1-year abstinence in smokers motivated to quit and participating in an intervention program. This 6-year retrospective observational cohort study was conducted in smokers who participated in an intervention program. The exhaled carbon monoxide (CO) was sequentially measured on day 1, 8, 15, and 22 of the intervention program. The primary outcome measure was smoking status at 1 year of follow-up. A total of 162 participants were enrolled and divided into a successful quit group (*n* = 52) and unsuccessful quit group (*n* = 110). Using a multivariate logistic regression analysis, we reported that the intention to quit (adjusted odds ratio [AOR] = 1.475, 95% confidence interval [CI] = 1.169–1.862, *P*-value = 0.001), varenicline use (AOR = 3.199, 95% CI = 1.290–7.934, *P* -value = 0.012) and the exhaled CO level on day 8 (AOR = 0.937, 95% CI = 0.885–0.992, *P*-value = 0.025) independently predicted 1-year smoking cessation. Moreover, the level of exhaled CO < 4.5 parts per million on day 8 significantly predict successful 1-year smoking cessation (area under curve 0.761, sensitivity 88.2%, and specificity 57.8%, *P*-value < 0.001). These independent predictors including intention to quit, varenicline use, and exhaled CO level on day 8, may help primary care physicians rearrange resources and refine the strategies for intervention programs to achieve a higher rate of long-term smoking cessation.

## Quitting smoking: Identifying predictors of success

Researchers in Taiwan identify key predictors that pinpoint people most likely to quit smoking successfully during intervention programs. Millions are spent each year supporting people to quit smoking. However, successful quitters remain in the minority, with only 9–35 per cent of those in intervention programs abstaining for at least a year. Hsin-Kuo Ko at Taipei Veterans General Hospital and co-workers identified key independent indicators of successful abstinence in 162 smokers attending an intervention program. Alongside having a high intention to quit and using varenicline medication, a potential predictor is having an exhaled carbon monoxide (CO) level of less than 4.5 parts-per-million by day 8 of the course. Exhaled CO is higher in smokers than in non-smokers. Measuring CO levels one week into courses may be a useful biomarker to identify those fully committed to quit.

## Introduction

Cigarette smoking is a modern-day epidemic that poses a substantial health burden and costs.^1,2^ In developed countries, cigarette smoking is the largest preventable cause of premature death and disability,^3–6^ and health risks associated with cigarette smoking may be reversed following a sufficient period of smoking cessation.^7,8^ Life-long smoking cessation is a critical public health objective while managing respiratory diseases in primary care, and smoking cessation is also crucial when managing all chronic diseases (e.g. hypertension and diabetes mellitus). Although both smoking cessation intervention programs and combined pharmacotherapy are effective in helping smokers quit, rates of 1-year sustained smoking cessation after the period of intervention are low (9.4–35.5%).^9–16^ Quitting tobacco use is difficult for numerous people. Therefore, identifying independent predictors of smoking cessation in smokers motivated to quit and participating in an intervention program is crucial in helping primary care physicians identify smokers who might need more intensive treatments and health-care resources.

In general population samples of smokers, previous attempts to quit, age of smoking initiation, motivation to quit, nicotine addiction levels, self-efficacy levels, and preparatory planning have been identified as predictors of smoking cessation.^17–25^ In smokers participating in an intervention program, gender, self-efficacy levels, use of bupropion, and preparatory planning have been identified as predictors of short-term smoking cessation.^22,26^ However, predictors of long-term smoking cessation (e.g., a 1 year period) in this population remain largely unknown. Moreover, there is no objective biomarker being able to be measured during an intervention program and utilized to predict long-term smoking cessation. Consequently, it is difficult for physicians to develop an effective strategy for smoking cessation intervention programs. To our knowledge, the level of carbon monoxide (CO) in exhaled air is higher in healthy smokers than in non-smokers, and measuring the exhaled CO level is an immediate, and non-invasive method for assessing the smoking status.^27,28^ Whether the objective biomarker, exhaled CO level, measured in smokers during the period of participation in a group-orientated intervention program can predict 1-year smoking cessation remains unclear.

We conducted a retrospective observational cohort study to determine the independent predictors of sustained 1-year smoking cessation in smokers participating in a group-oriented smoking cessation program. We particularly focused on the role of exhaled CO measured during an intervention program in predicting long-term smoking cessation. The results of our study may help physicians to develop effective strategies for intervention program to increase the rate of long-term smoking cessation in primary respiratory care.

## Results

### Demographic characteristics of study participants

During this 6-year study period, 202 consecutive smokers who participated in the smoking cessation program were reviewed. A total of 40 smokers were excluded because of (1) quitting smoking before the courses (*n* = 8), (2) failure to obtain complete information on their smoking status at 1, 3, 6, and 12 months of follow-up (*n* = 22), (3) failure to complete all smoking cessation courses (*n* = 10). In total, 162 smokers including 151 men and 11 women with a mean age 50.1 years (±17.1) were enrolled and analyzed in this study. The mean age of smoking initiation was 21.8 (±10.2) years, duration of smoking 30.2 (±15.8) years, and number of cigarette packs smoked per day 29.3 (±25.9). In total, 46 of 162 participants (28.4%) used varenicline for more than 8 weeks. The overall rates of successful smoking cessation at the first, third, and sixth month, and 1 year of follow-up were 38.8, 37.0, 36.6, and 32.1%, respectively.

### Factors associated with the 1-year sustained smoking cessation

All participants (*n* = 162) were divided into the unsuccessful quit (*n* = 110) and successful quit (*n* = 52) groups according to their smoking cessation status at 1 year of follow-up. The successful quit smokers were significantly associated with a higher mean score on the Likert scale of intention to quit (7.8 ± 1.9 vs. 6.1 ± 1.9, *P*-value < 0.001), and with a higher percentage of varenicline use (46.2 vs. 20.0%, *P*-value = 0.001) compared with the unsuccessful quit smokers (Table 1).

**Table 1:** Clinical characteristics of study participants according to the smoking status at 1 year of follow-up

Variables	Total	Unsuccessful quit	Successful quit	*P*-value
	(*n* = 162)	(*n *= 110)	(*n *= 52)	
Age (yr)	50.1 ± 17.1	50.8 ± 16.6	48.9 ± 18.2	0.532
Body mass index (kg/m^2^)	24.3 ± 3.3	24.3 ± 3.4	24.3 ± 3.1	0.708
Male	151 (93.2)	102 (92.7)	49 (94.2)	1.000
Education (yr)	13.5 ± 2.1	13.4 ± 2.2	13.7 ± 1.8	0.653
Being married	119 (73.5)	84 (76.4)	35 (67.3)	0.255
Age at starting smoking (yr)	21.8 ± 10.2	22.3 ± 10.5	20.8 ± 9.4	0.112
Duration of cigarette smoking (yr)	30.2 ± 15.8	30.7 ± 15.1	28.7 ± 17.3	0.504
Smoking (pack-yr)	29.3 ± 25.9	30.7 ± 15.1	28.7 ± 17.3	0.504
Comorbidity				
Asthma	7 (4.3)	5 (4.5)	2 (3.8)	1.000
COPD	26 (16.0)	19 (17.3)	7 (13.5)	0.649
Hypertension	35 (21.6)	24 (21.8)	11 (21.1)	1.000
Diabetes mellitus	17 (10.5)	11 (10)	6 (11.5)	0.787
Addiction level	4.5 ± 2.0	4.6 ± 1.9	4.3 ± 2.2	0.172
Intention to quit scale	6.6 ± 2.0	6.1 ± 1.9	7.8 ± 1.9	<0.001
Varenicline use	46 (28.4)	22 (20.0)	24 (46.2)	0.001
Pulmonary function test				
FEV1/FVC (%)	77.4 ± 10.0	77.1 ± 10.6	77.8 ± 8.7	0.726
FEV1(liter)	3.1 ± 0.8	3.1 ± 0.8	3.2 ± 0.8	0.556
FEV1 (% predicted)	96.7 ± 16.6	96.5 ± 17.1	97.0 ± 15.5	0.989
FEF 25–75 (% predicted)	71.4 ± 30.1	72.4 ± 31.3	69.4 ± 30.4	0.654
FEF 25–75% predicted<80%	104 (64.2)	67 (60.9)	37 (71.2)	0.291
Exhaled CO level (ppm)				
On day 1 (*n* = 124)	17.5 ± 13.5	18.9 ± 14.3	14.9 ± 11.6	0.107
On day 8 (*n* = 121)	11.7 ± 11.8	14.7 ± 13.0	6.6 ± 7.3	<0.001
On day 15 (*n* = 107)	10.5 ± 11.2	13.1 ± 12.0	6.0 ± 7.9	<0.001
On day 22 (*n* = 69)	9.1 ± 11.0	11.5 ± 12.8	5.4 ± 6.3	0.010

Data are reported as the mean ± standard deviation or number (percentage)

*CO* carbon monoxide, *COPD* chronic obstructive pulmonary disease, *FEF* forced expiratory flow, *FEV1* forced expiratory volume in one second, *FVC* forced vital capacity, *ppm* parts per million

**Fig. 1:**
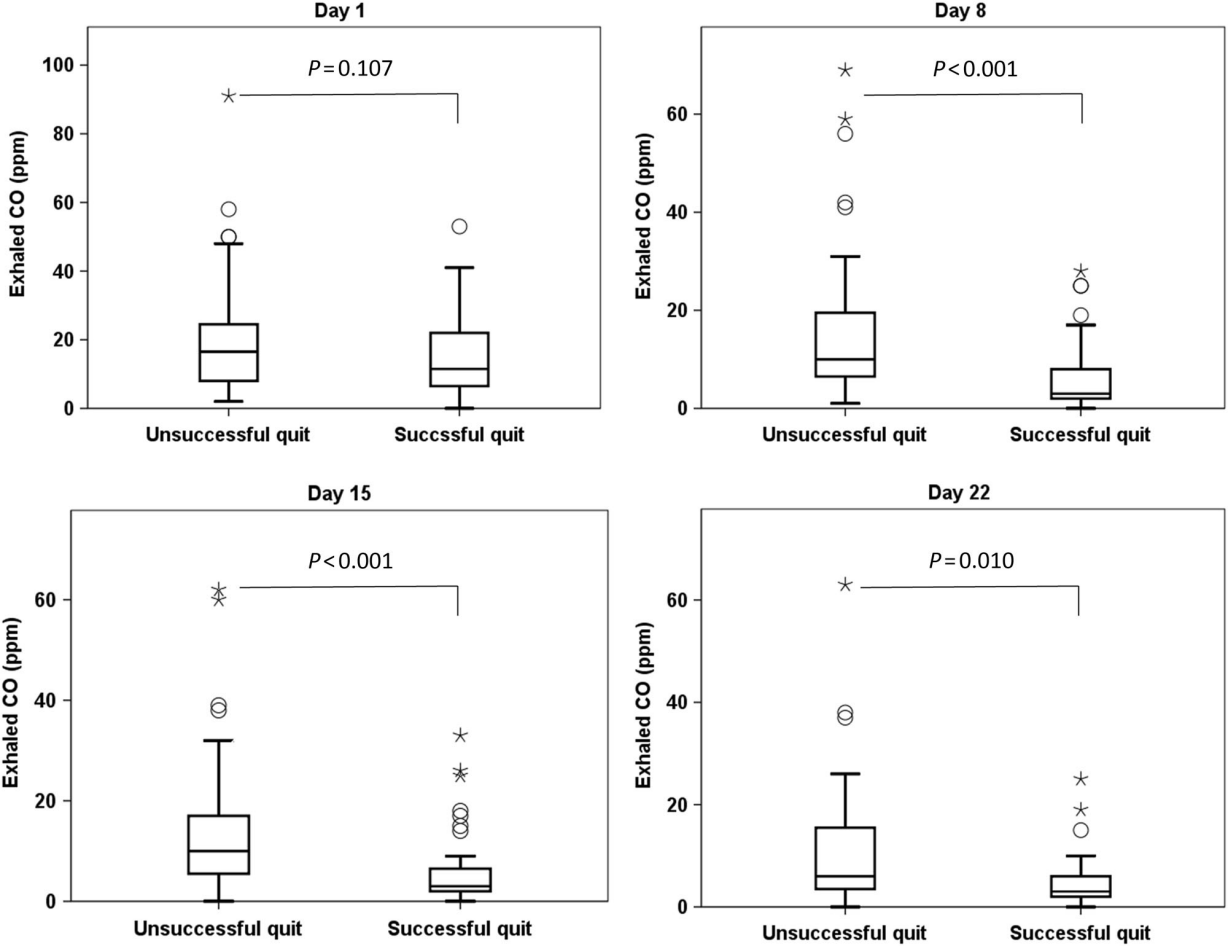
Distribution of exhaled carbon monoxide on day 1 (*n* = 124), day 8 (*n* = 121), day 15 (*n* = 107), and day 22 (*n* = 69) in smokers who quit unsuccessfully and those who quit successfully

**Fig. 2:**
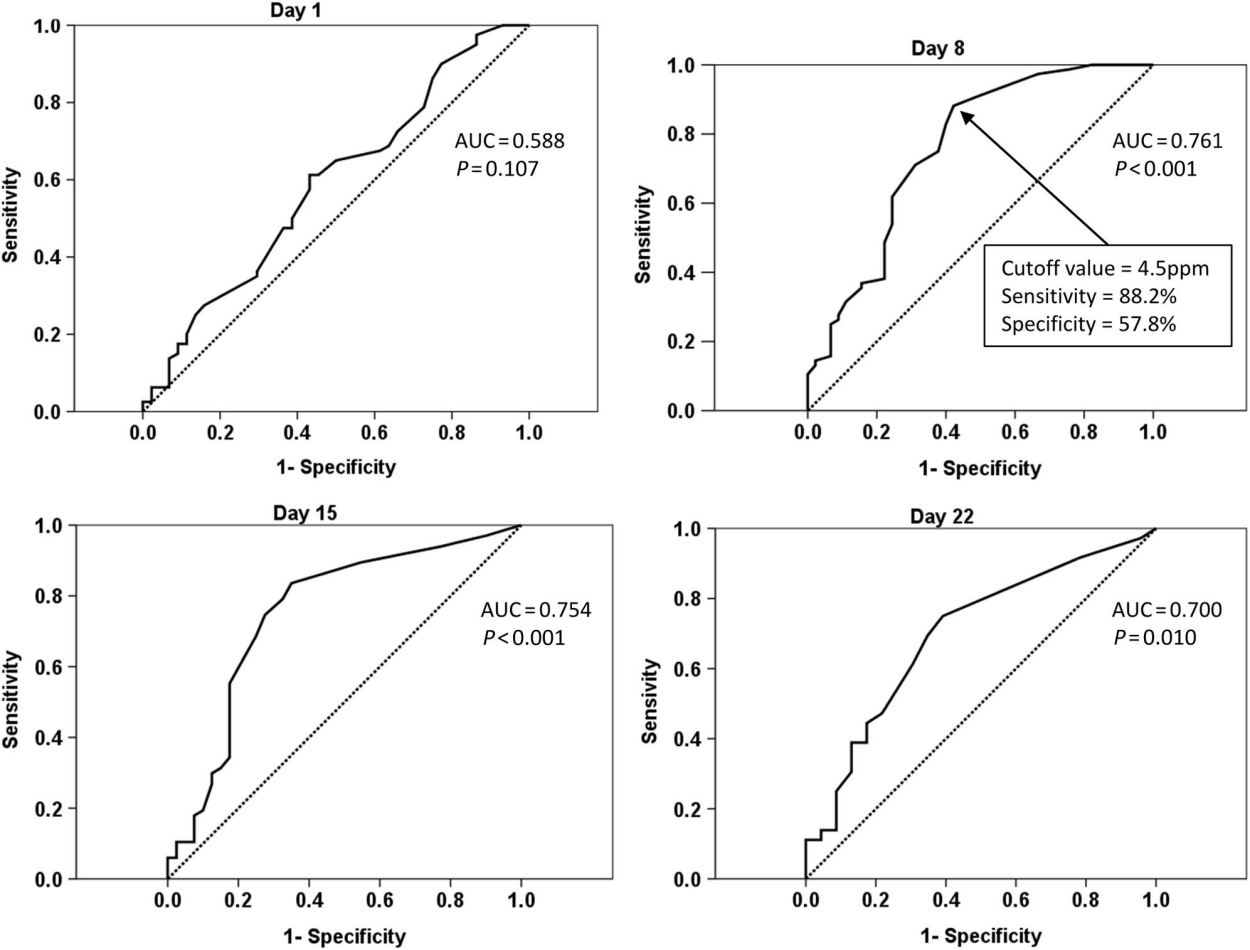
Receiver operating characteristic curve analysis for exhaled carbon monoxide on day 1, day 8, day 15, and day 22 in study participants for determining the cutoff level for exhaled carbon monoxide with the highest sensitivity and specificity

The successful quit smokers exhibited lower levels of exhaled CO on day 8 (6.6 ± 7.3 parts per million [ppm] vs. 14.7 ± 13.0 ppm, *P-*value < 0.001), day 15 (6.0 ± 7.9 ppm vs. 13.1 ± 12.0 ppm, *P*-value < 0.001), and day 22 (5.4 ± 6.3 ppm vs. 11.5 ± 12.8 ppm, *P-*value < 0.001) compared with the unsuccessful quit smokers (Table 1, Fig. 1). The ROC curves were estimated for exhaled CO levels on day 1, day 8, day 15, and day 22 (Fig. 2). A cutoff level for exhaled CO of 4.5 ppm on day 8 exhibited the highest sensitivity and specificity in differentiating successful 1-year smoking cessation from unsuccessful cessation (area under curve = 0.761, sensitivity = 88.2%, specificity = 57.8%, *P-*value < 0.001). Therefore, the exhaled CO level on day 8 was used as a potential predictor in multivariate logistic regression analysis. No significant difference between the two groups was observed with respect to age, education level, baseline smoking status, comorbidity, addiction level, and pulmonary function values.

### Independent predictors of 1-year sustained smoking cessation

The predictors of successful 1-year smoking cessation in participants were further analyzed using multivariate logistic regression (Table 2). Significantly independent predictors of 1-year successful cessation were intention to quit (adjusted odds ratio [AOR] = 1.475; 95% confidence interval [CI] = 1.169–1.862, *P*-value = 0.001), varenicline use (AOR = 3.199, 95% CI = 1.290–7.934, *P-*value = 0.012), and the exhaled CO level on day 8 (AOR = 0.937, 95% CI = 0.885–0.992, *P-*value = 0.025). Intention to quit, varenicline use, and the exhaled CO level on day 8 were also the independent predictors of successful smoking cessation after 1, 3, and 6 months of follow-up.

**Table 2:** Multivariate logistic regression analysis of potential predictors for smoking cessation at 1, 3, 6 month and 1 year of follow-up in the study subjects

	1-month successful quit	3-month successful quit	6-month successful quit	1-year successful quit
Intention to quit	1.695	1.636	1.619	1.475
	(1.313–2.189)**	(1.299–2.110)**	(1.266–2.072)**	(1.169–1.862)*
Varenicline use	5.975	6.278	4.496	3.199
	(2.128–16.777)*	(2.270–17.367)*	(1.688–11.969)*	(1.290–7.934)*
Exhaled CO level onday 8	0.928	0.929	0.929	0.937
	(0.878–0.981)*	(0.877–0.984)*	(0.877–0.985)*	(0.885–0.992)*

Data are reported as adjusted odds ratio (95% confidence interval)

*CO* carbon monoxide

*P-value  <  0.05, **P-value  <  0.001

## Discussion

### Main findings

In the present study, we found that intention to quit, varenicline use, and the exhaled CO level on day 8 to be independent predictors of 1-year sustained smoking cessation in motivated smokers who participated in a group-oriented intervention program. Meanwhile, a cutoff level of 4.5 ppm for exhaled CO levels on day 8 that exhibited sensitivity of 88.2% and specificity of 57.8% could be considered as a useful biomarker in differentiating successful 1-year smoking cessation from unsuccessful cessation.

### Interpretation of findings in relation to previously published work

Common predictors of smoking cessation in clinical practice have been widely investigated and comprehensively reviewed.^29^ Several blood biomarkers have been reported to indicate smoking status, including nicotine, cotinine, or thiocyanate levels in the plasma or urine.^30^ Following cigarette smoke inhalation, CO displace oxygen in the erythrocyte to form carboxyhemoglobin (COHb). CoHb has a half-life of about 5–6 h,^31^ and CO in alveolar air after breath holding is in equilibrium with the concentration of COHb in the blood.^32^ Middleton et al. report a significant correlation between breath CO and the number of cigarettes smoked in the last 24 h, and several studies have reported a breath CO level more than 6–8 ppm giving an appropriate sensitivity and specificity/selectivity to detect a smoker in outpatient clinics.^27,28,30,33^ In past, measuring the biomarker, exhaled CO level, was considered as an immediate, and non-invasive method for assessing smoking status and differentiating healthy smokers from nonsmokers.^27,28^ To our knowledge, there is no study reporting serial exhaled levels of CO measured in an intervention program to demonstrate the role of exhaled CO in predicting the long-term outcome of smoking cessation. In the present study, the levels of exhaled CO on day 1, day 8, day 15, and day 22 were sequentially measured during the smoking cessation program. We observed that exhaled CO levels on day 1 of smoking cessation program were not significantly different between successful and unsuccessful quit groups. A cutoff level of 4.5 ppm for exhaled CO levels on day 8 exhibited a sensitivity of 88.2% and a specificity of 57.8% to predict successful 1-year smoking cessation. Notably, intention to quit when attending a smoking cessation program was also an independent predictor of long-term smoking cessation. These findings of our study suggested that intention to quit was an early predictor and to measure exhaled CO level in smokers after the intervention program starts may well be a function of those who quit most rapidly and persistently. Results of the study can also be interpreted that people who are smoke free at least one week increase their success rate of smoking cessation.

Several factors have influence on the metabolism of COHb and exhaled levels of CO. The half-life of COHb can be influenced by gender, physical activity, and ventilation rate,^34–36^ and impaired lung function affects the relationship between expired air CO and COHb.^32^ Additionally, high levels of exhaled CO can be caused by environmental pollution, occupational exposure, passive smoking, active smoking, and airway inflammation in asthmatic lungs.^37^ In the current study, we enrolled subjects with a diagnosis of COPD and asthma (33/162, 20%) and reported the data of baseline lung function. We found that there was not statistical difference between unsuccessful and successful quit group among gender, data of lung function and the diagnosis of obstructive lung diseases. This finding may further confirm the generalization of applying exhaled CO level in predicting the outcome of long-term smoking cessation.

In total, 70% of smokers report that they want to quit;^38^ however, annually only 2–7% of smokers succeed.^39,40^ Pharmacotherapies and smoking cessation programs have been recommended by the World Health Organization as effective methods for motivated smokers to achieve higher rates of smoking cessation.^2^ A combination of sustained-release bupropion and a nicotine patch was studied by Jorenby et al. in 893 smokers. Treatment with a combination of sustained-release bupropion and a nicotine patch yielded a significantly higher rate of smoking cessation at 12 months than did treatment with sustained-release bupropion, or a nicotine patch alone and a placebo alone (35.5, 30.3, 16.4 vs. 15.6%, respectively).^15^ Varenicline, an agonist of the α_4_β_2_ neuronal nicotinic acetylcholine receptor, is commonly used for facilitating smoking cessation. Koegelenberg et al. enrolled 446 smokers and blindly randomized them for either combination therapy with varenicline and a nicotine patch or varenicline monotherapy alone.^41^ The combination therapy exhibited significantly higher rates of continuous smoking cessation at 6 months (49.0 vs. 32.6%) than the varenicline monotherapy did. Ebbert et al. analyzed combination therapy with varenicline and sustained-release bupropion in a randomized, blinded, controlled clinical trial and reported that the combination therapy did not significantly increase the rate of 1-year smoking cessation compared with varenicline monotherapy (30.9 vs. 24.5%).^42^ Additionally, smoking cessation programs with financial incentives have been studied to determine whether the interventions increase the rate of sustained smoking cessation compared with usual care.^9,10^ Halpern et al. concluded that the programs with financial incentives exhibited significantly higher rates of smoking cessation at 6 months than usual care did (9.4–16.0 vs. 6.0%).^9^ In the present study, the fee for the smoking cessation courses and varenicline use was covered by the Health Promotion Administration, Ministry of Health and Welfare, Taiwan. Physicians of our Department prescribed varenicline, and there was no participant receiving bupropion or nicotine replacement therapy (NRT) simultaneously during the period of smoking cessation program. Despite a low percentage of participants receiving a pharmacotherapy (varenicline use in 28.4% of study participants) and no financial incentive being offered in the smoking cessation program, a relatively high rate of 1-year smoking cessation (32.1%) was reported as compared with previous studies.^9,10,41,42^ Furthermore, the present study similarly showed that varenicline use was an independent predictor of long-term smoking cessation. The results of the present study and previous studies indicate that varenicline therapy is an effective combined pharmacotherapy during smoking cessation programs for increasing long-term smoking cessation.

### Strength and limitations of this study

According to our review of relevant research, the present study is aimed to sequentially measure exhaled CO levels in smokers during a smoking cessation program and identify the biomarker of exhaled CO levels on day 8 as an independent predictor of long-term smoking cessation.

Our study has several limitations. First, it was a retrospective observational study. The measurement of exhaled CO is based on the willingness to accept and the number of smokers with measuring CO at each measurement point is declined. Some data are likely to be missing and cannot be recovered. Second, some participants were diagnosed with chronic obstructive pulmonary disease and asthma. The effect of chronic airway inflammation on the levels of exhaled CO in these smokers remains unclear.^37,43^ Third, self-reported smoking status at the time points of measuring exhaled CO is not recorded. The relationship between smoking cessation and exhaled CO levels after the program starts remains undefined. Finally, the results were originated from a population of motivated smokers who participated in a smoking cessation program. Whether the predictors of our study are applicable to all smokers and these findings can be generalized to all primary care patients remains unknown.

### Implications for future research, policy, and practice

We reported that levels of exhaled CO were not only the indicator of current smoking status but also an independent predictor of long-term smoking cessation in motivated smokers. Meanwhile, a cutoff level of 4.5 ppm of exhaled CO on day 8 can be used to differentiate successful 1-year smoking cessation from unsuccessful cessation (a sensitivity = 88.2%, and specificity = 57.8%). The biomarkers of exhaled CO may provide primary care physicians with the critical and immediate information required to rearrange resources, change policy and refine the strategies for intervention programs to achieve a higher rate of long-term smoking cessation. Whether our findings could actually assist in the clinician’s decision making, and the concept of exhaled CO levels as the objective biomarker to rearrange the medical resource deserve to be prospectively investigated and validated in future researches.

## Conclusions

In motivated smokers participating in a group-oriented smoking cessation program, intention to quit, varenicline use, and the exhaled CO level on day 8 independently predict 1-year smoking cessation. A cutoff level of 4.5 ppm for exhaled CO on day 8 of the intervention program exhibits the highest sensitivity and specificity in differentiating 1-year successful smoking cessation and unsuccessful cessation. Main conclusion is that intention to quit and use of varenicline is the strongest predictor for 1-year successful quit and this is also in agreement with other studies.

## Methods

### Study design and smoking cessation intervention program

A retrospective observational cohort study was conducted at Taipei Veterans General Hospital, a tertiary medical center in Taiwan. Regular smokers were targeted for recruitment, and no financial incentives were offered for participating in the group-oriented intervention program. A multifaceted recruitment scheme was used to enroll hospital employees, or their relatives and friends, and participants through advertisements on the website of Taipei Veterans General Hospital. The same smoking cessation program over the study period and instructors of the smoking cessation courses were approved by the Health Promotion Administration, Ministry of Health and Welfare, Taiwan, ROC. The 22-day smoking cessation program comprised four smoking cessation courses (3 h per course, 12 h in total). Medications for smoking cessation, sustained-release bupropion and varenicline, and NRT were prescribed by pulmonologists of the Department of Chest Medicine according to the needs of participants during the period of the smoking cessation courses. The study was approved by the Institutional Ethical Review Board of Taipei Veterans General Hospital (VGHTPE-IRB No. 2015-08-001CE), and Informed consent was waived under the approval of the VGHTPE-IRB according to the institutional guideline for a retrospective observational study. Methods were performed in accordance with relevant regulations and guidelines.

### Study participants

Smokers who attempted to quit smoking and participated in the intervention program for smoking cessation at the Department of Chest Medicine of Taipei Veterans General Hospital between July 2008 and June 2014 were reviewed. Participants were excluded, if (1) they had stopped cigarette smoking within 1 month before participating in the smoking cessation courses, (2) they were not able to complete the smoking cessation courses, and (3) the information on their smoking status at 1, 3, 6, and 12 months of follow-up was not obtained. The enrolled smokers were divided into successful and unsuccessful 1-year sustained quit groups according to their smoking status 12 months after completing all four smoking cessation courses.

### Baseline measurements

The departmental log was used to identify the participants, and the following demographic information was collected through a careful chart review by using a specifically designed data sheet: age, sex, marital status, height, body weight, education level, comorbidity, respiratory system symptoms, and the history of cigarette smoking (numbers and years). Medications, namely SR bupropion and varenicline, and NRT were reviewed carefully and recorded. The addiction level was measured using the abbreviated Fagerström Test for Cigarette Dependence (0 = not addicted, 10 = highly addicted).^44,45^ Intention to quit smoking was measured on a 10-point likert scale by asking the participant whether or not he or she intended to quit smoking (1 = very certainly not; 10 = very certainly yes).

### Follow-up measurements

A pulmonary function test was conducted within 1 week after participation in the smoking cessation program. The exhaled CO levels of the participants were measured using a MicroCO meter (CareFusion, CA, USA) on day 1, day 8 and day 15, and day 22 of smoking cessation program. The smoking status at the 12th month of follow-up after the completion of the smoking cessation courses was set as the primary outcome measure. Furthermore, the self-reported smoking status at the first month, third month, sixth month, and twelfth month of follow-up by interviewing the participants by phone. Successful 1-year smoking cessation was achieved when a participant reported not smoking since the completion of the smoking cessation courses.

### Statistical analysis

All data were expressed as the mean ± standard deviation or as a percentage (%). Categorical variables were compared using the chi-squared test or Fisher’s exact test. Regarding numerical variables, the Mann–Whitney *U* test was used for non-normally distributed variables, and the independent *t*-test was used for normally distributed variables. Variables exhibiting significant differences between the two groups (*P*-value < 0.05) were subjected to multivariate forward stepwise logistic regression analysis to determine factors independently associated with successful smoking cessation at 1, 3, 6 months, and 1 year. Receiver operating characteristic (ROC) curve analysis was used to identify optimal cutoff points for creating dichotomous variables. All statistical analyses were conducted using SPSS, version 19.0 (SPSS Inc., Chicago, IL, USA). The results were considered significant at a two-sided *P-*value<0.05.

### Data availability statement

The data that support the findings of this study are available from the corresponding author upon reasonable request.
